# Global, regional, and national burden of interstitial lung disease in middle-aged and older adults from 1990 to 2021 with projections to 2050: a systematic analysis of the 2021 Global Burden of Disease Study

**DOI:** 10.3389/fmed.2025.1671638

**Published:** 2025-10-14

**Authors:** Long Chen, Jiuhe Wang, Jinglian Qu

**Affiliations:** ^1^Guizhou University of Traditional Chinese Medicine, Guiyang, China; ^2^Department of Heart Diseases, Inner Mongolia Autonomous Region Hospital of Traditional Chinese Medicine, Hohhot, China

**Keywords:** disease epidemiology, interstitial lung disease, global disease burden, frontal analysis, forecast

## Abstract

**Background:**

Interstitial lung disease (ILD) constitutes a significant global health challenge. However, existing epidemiological studies predominantly focus on worldwide and regional distribution patterns, while a substantial knowledge gap persists regarding the epidemiological characteristics of ILD among middle-aged and older adults (50–74 years). Therefore, this investigation comprehensively evaluates the disease burden of interstitial lung disease across global, regional, and national levels within the specified population.

**Methods:**

Utilizing data from the 2021 Global Burden of Disease (GBD) study, we employed key age-standardized metrics prevalence rate (ASPR), incidence rate (ASIR), death rate (ASDR), and disability-adjusted life years (DALYs) rate-to quantify the disease burden. Temporal patterns from 1990 to 2021 were assessed using Joinpoint regression analysis, deriving both the annual percentage change (APC) and (AAPC). Frontier analysis benchmarked national regional performance against optimal achievers and projected disease trajectories over the next 25 years.

**Results:**

In 2021, a global total of 2,524,796 prevalent ILD cases occurred among adults aged 50–74 years, concurrent with sustained increases in ASPR, ASIR, ASDR, and DALY rates. Andean Latin America exhibited the highest ASIR, ASDR, and DALY rates, while High-Income Asia Pacific registered the peak ASPR. Uniform upward trajectories in prevalence, incidence, mortality, and DALYs were consistently observed across all age groups during 1990–2021. Joinpoint regression analysis revealed that while ASPR and ASIR increased from 1990 to 2010 but subsequently decreased from 2010 to 2021, conversely, ASDR and DALYs increased from 1990 to 2011 before declining after 2011, frontier analysis highlighted Canada, Ireland, the United States, Japan, and the United Kingdom as nations with significant improvement potential. By 2050, we project that the burden of ILD among middle-aged and elderly populations will remain substantial, with only a slight decline in the ASPR.

**Conclusion:**

Globally, the burden of ILD in middle-aged and older populations exhibits a progressive increase; however, marked geographical variations are evident. The Andean Latin American region exhibits particularly elevated burden rates. Consequently, multifaceted interventions-including air quality improvement, tobacco cessation promotion, enhanced availability of high-resolution computed tomography (HRCT), and increased public health awareness of ILD-represent essential strategies to address this burden.

## 1 Introduction

Interstitial lung disease (ILD) encompasses a spectrum of disorders unified by pathological inflammation and fibrosis within the pulmonary interstitium. *Since* the pathological involvement extends to alveolar walls, alveolar spaces, *as well as* adjacent small and large airways, it is *therefore* alternatively termed diffuse parenchymal lung disease (1). The median survival period is merely 2–3 years (2). Clinically, ILD manifests predominantly as dry cough and progressive dyspnea. These symptoms ultimately lead to irreversible pulmonary damage, thereby compromising quality of life and inevitably culminating in respiratory failure. Consequently, this disease exhibits significantly elevated morbidity and mortality rates (3). ILD typically presents with an insidious onset and diverse, complex etiologies, consequently posing significant diagnostic challenges. Furthermore, current therapeutic options remain limited, often demonstrating suboptimal efficacy and substantial adverse drug reactions (4). Current therapeutic objectives for ILD primarily center on slowing disease progression, improving patients' quality of life, and extending survival. However, for select advanced-stage patients, lung transplantation remains the only definitive intervention capable of enhancing both survival duration and quality of life. Consequently, ILD is established as a significant global medical challenge, representing a critical frontier in respiratory medicine that imposes substantial societal burdens (5).

In recent decades, the prevalence of interstitial lung disease ILD has exhibited a persistent upward trajectory, which is particularly pronounced in aging societies (6). This trend extends beyond direct healthcare expenditures to encompass substantial indirect costs associated with productivity losses and disability, thus amplifying the socioeconomic impact. Consequently, ILD remains a persistent global public health challenge. From 1990 to 2019, global ILD incidence increased by 118.6%, while mortality rates and death counts rose substantially more, by 166.63 and 122.87%, respectively (7). Current epidemiological data indicate that the prevalence of ILD ranges from 6.3 to 71 cases per 100,000 population, whereas the annual incidence varies between 1.0 and 31.5 cases per 100,000 person-years (2). In French studies, however, the prevalence rate was reported as 97.9 per 100,000 population, with an annual incidence rate of 19.4 cases (8). In contrast, idiopathic pulmonary fibrosis (IPF) represents the predominant form of ILD in both Europe and North America (9). In Asia, however, hypersensitivity pneumonitis is predominantly observed. Key Improvements and Reasoning (10), Consequently, these epidemiological findings underscore the substantial heterogeneity of ILD and highlight its significant public health challenge globally.

As a manageable and significant public health concern, ILD therefore exhibits epidemiological characteristics critical for informing health strategy formulation. Consequently, given the escalating trend of population aging, there is an urgent need to obtain global ILD epidemiological data with a specific focus on middle-aged and elderly populations. Such research will thus deepen the understanding of ILD prevalence among this demographic, thereby assisting healthcare decision-makers, epidemiologists, and clinicians in optimizing resource allocation and implementing targeted public health interventions.

## 2 Methods

### 2.1 Data sources

The GBD 2021 employs a systematic framework to comprehensively evaluate 371 diseases and injuries, synthesizing diverse data sources—specifically vital registration systems, verbal autopsy records, population censuses, household surveys, disease registries, and clinical encounter datasets (11). The following address deals with access to the information table and representation of effects: https://vizhub.healthdata.org/gbd-results. Consistent with the GBD framework, ILD comprises chronic respiratory disorders characterized by pulmonary scarring and/or inflammation, which ultimately impair lung function and reduce oxygenation capacity (12). According to the 10th revision of the International Classification of Diseases (ICD-10), J84 specifically refers to “Other Interstitial Lung Diseases” (13, 14). This study extracted data on age-standardized metrics prevalence rate (ASPR), incidence rate (ASIR), death rate (ASDR), and disability-adjusted life years (DALYs) related to ILD in middle-aged and older adults from the GBD 2021 project, covering the period from 1990 to 2021 across 204 countries and territories. The analyses were stratified by age, sex, geographical region, country, and SDI. DALYs represent the sum of years lived with disability (YLD) and years of life lost (YLL). YLD was calculated by multiplying the prevalence of each sequela by its disability weight, whereas YLL was derived by multiplying the number of deaths by the standard life expectancy at the age of death. All rates were age-standardized using the GBD global reference population (15). In accordance with the standard GBD classification, the study population was categorized into five age groups: 50–54, 55–59, 60–64, 65–69, and 70–74 years. This age range was selected to balance sample representativeness, clinical relevance, and methodological rigor, thereby enhancing the internal validity and interpretative reliability of the study findings. GBD 2021 can provide estimated values for all indicators along with their uncertainty intervals (UI) (16).

### 2.2 Joinpoint regression analysis

This analysis applied Joinpoint Regression (Version5.2.0) to quantify temporal patterns in ASPR, ASIR, ASDR rates, and DALYs for interstitial lung disease among older adults. The Joinpoint software employs a grid search method as its modeling strategy to identify potential joinpoints and calculates the sum of squared errors (SSE) and mean squared error for each scenario. Consequently, a maximum of five joinpoints was permitted in this study. The Joinpoint model yielded annual percentage change (APC) estimates and corresponding 95% confidence intervals (CI), enabling computation of average annual percentage change (AAPC) values (17), each *P* value was determined using the Monte Carlo method. A *P* value < 0.05 was considered to be statistically significant (18).

### 2.3 Frontier analysis

To evaluate the relationship between the burden of ILD in middle-aged and elderly populations and socio-demographic development levels, we employed frontier analysis, constructing frontier models for ASPR, ASDR, and DALYs using the SDI. Unlike traditional regression models that describe variable relationships or predict outcomes, this study employs frontier analysis and other sophisticated statistical methods to investigate the non-linear association between the Sociodemographic Index (SDI) and disease burden, while identifying multidimensional driving factors. The core of this technique lies in establishing a theoretically achievable minimum burden value as an optimal performance benchmark, based on each country region's current development level. By quantifying the gap between actual burden and potential minimal burden, this quantification therefore identifies priority areas for improvement. To ensure analytical robustness, we computed average DALYs for each SDI quintile using 1,000 bootstrap samples. Consequently, by measuring the vertical distance between actual values and the frontier benchmark for each country region in 2021, we systematically assessed their improvement potential (19).

### 2.4 Projection model development

The present study implemented the Bayesian age-period-cohort (BAPC) modeling framework to generate future projections. In contrast to multiple linear power models, however, the BAPC model offers distinct advantages, thereby generating more plausible projection outcomes. Critically, existing studies have confirmed that the BAPC model provides well-calibrated probabilistic projections while also maintaining reasonably narrow prediction intervals (20). Consequently, the categorical settings for age groups and periods within the model align with the standard age-period-cohort framework. The analysis was subsequently performed using the BAPC package within the R statistical environment (21).

### 2.5 Ethics statement

This study did not involve animal or human subjects, nor did it utilize any potentially identifiable human images or data.

### 2.6 Statistical analysis

All Statistical analyses were conducted with R software (version 4.1.3), GraphPad Prism (v10.0), and the Joinpoint Regression Program (v5.2.0).

## 3 Results

### 3.1 Disease burden of ILD

#### 3.1.1 Global trends

The global burden of ILD among middle-aged and elderly populations continued to escalate in 2021. The total prevalent cases reached 2,524,796 (95% UI: 2,029,172–3,101,383), significantly exceeding the 1990 figure of 1,110,560 (95% UI: 873,162–1,386,472). Consequently, the ASPR rose from 147.0/100,000 (95% UI: 115.76–183.45) in 1990 to 153.7/100,000 (95% UI: 123.55–188.84) in 2021, reflecting an estimated average annual percentage change (EAPC) of 0.3 (95% CI: 0.2–0.3; Table 1, Supplementary Table S2). Similarly, the incidence of ILD demonstrated an upward trajectory. Globally, incident cases rose to 225,572 (95% UI: 143,247–318,422) in 2021, substantially surpassing the 1990 count of 85,177 (95% UI: 51,853–124,788). Correspondingly, the age-standardized incidence rate (ASIR) increased from 11.20 per 100,000 (95% UI: 6.80–16.40) to 13.70 per 100,000 (95% UI: 8.70–19.40), exhibiting an EAPC of 0.80 (95% CI: 0.7–0.9), notably the highest among all indicators (Table 1, Supplementary Table S2). Mortality-related metrics concomitantly increased. Deaths climbed to 72,574 (95% UI: 58,054–86,455) in 2021, marking a significant rise from 27,836 (95% UI: 21,355–36,277) in 1990. Accordingly, the ASDR increased from 3.7 per 100,000 (95% UI: 2.9–4.9) to 4.42 per 100,000 (95% UI: 3.5–5.3), with an EAPC of 0.7 (95% CI: 0.6–0.8; Table 1, Supplementary Table S2). Disability-adjusted life year (DALY) analysis further confirmed the growing overall disease burden. Global DALYs reached 2,105,047 (95% UI: 1,737,325–2,477,355) in 2021, considerably higher than the 1990 value of 849,373 (95% UI: 669,006–1,077,398). Consequently, the age-standardized DALY rate increased from 113.00 per 100,000 (95% UI: 89.10–143.20) to 128.20 per 100,000 (95% UI: 105.80–150.80), yielding an EAPC of 0.50 (95% CI: 0.40–0.60; Table 1, Supplementary Table S2).

**Table 1:** Prevalence, incidence, death, disability-adjusted life years (DALYs), disability-adjusted rate (ASR), and the annual percentage change in population (EAPC) of interstitial lung disease (ILD) among the Middle-Aged and Older Adults in the global and regional contexts in 2021, as well as from 1990 to 2021.

**Location**	**Prevalence cases (95%UI)**	**ASR per 100,000 (95%UI)**	**EAPC (95%CI)**	**Incidence cases (95%UI)**	**ASR per 100,000 (95%UI)**	**EAPC (95%CI)**
Global	2,524,796 (2,029,172, 3,101,383)	153.73 (123.55, 188.84)	0.26 (0.18, 0.34)	225,572 (143,247, 318,422)	13.70 (8.70, 19.40)	0.80 (0.68, 0.91)
High SDI	1,068,326 (869,935, 1,300,365)	301.29 (244.69, 367.50)	0.39 (0.30, 0.48)	91,851 (58,388, 128,962)	26.00 (16.50, 36.60)	0.99 (0.85, 1.13)
High-middle SDI	435,263 (353,202, 527,902)	114.53 (92.81, 139.05)	0.40 (0.27, 0.53)	33,672 (21,455, 47,014)	8.90 (5.70, 12.50)	1.34 (1.12, 1.55)
Middle SDI	561,029 (443,505, 698,758)	104.82 (83.01, 130.38)	0.58 (0.47, 0.68)	50,824 (31,869, 72,473)	9.50 (6.00, 13.50)	1.06 (0.92, 1.20)
Low-middle SDI	370,965 (292,669, 463,846)	133.20 (105.18, 166.38)	0.33 (0.29, 0.37)	39,678 (25,045, 56,839)	14.20 (9.00, 20.40)	0.29 (0.27, 0.32)
Low SDI	87,831 (69,171, 109,924)	89.42 (70.54, 111.70)	0.13 (0.10, 0.17)	9,453 (5,963, 13,524)	9.70 (6.10, 13.80)	0.15 (0.13, 0.18)
East Asia	439,107 (344,718, 550,364)	98.70 (77.54, 123.65)	0.75 (0.52, 0.97)	33,585 (20,333, 48,782)	7.50 (4.60, 11.00)	1.90 (1.47, 2.33)
Central Asia	17,700 (14,711, 21,035)	105.53 (87.99, 125.21)	−0.02 (−0.27, 0.24)	1,451 (952, 2,024)	8.70 (5.80, 12.10)	0.08 (−0.18, 0.33)
South Asia	495,196 (385,943, 623,698)	172.48 (134.47, 217.11)	0.21 (0.17, 0.25)	54,881 (34,345, 79,613)	19.10 (12.00, 27.70)	0.16 (0.14, 0.18)
Southeast Asia	81,840 (64,120, 101,944)	61.18 (48.13, 76.00)	0.89 (0.87, 0.90)	6,916 (4,242, 9,937)	5.10 (3.10, 7.30)	0.66 (0.64, 0.69)
High-income Asia Pacific	331,655 (269,694, 404,709)	474.10 (384.13, 579.86)	0.23 (0.11, 0.34)	27,974 (17,916, 38,888)	41.30 (26.40, 57.60)	0.76 (0.57, 0.95)
Oceania	2,063 (1,719, 2,437)	133.72 (111.71, 157.56)	0.38 (0.33, 0.42)	160 (102, 222)	10.20 (6.60, 14.20)	0.44 (0.39, 0.49)
Australasia	16,968 (13,913, 20,518)	181.42 (148.10, 220.15)	1.44 (1.24, 1.63)	1,976 (1,343, 2,658)	21.10 (14.30, 28.50)	2.07 (1.82, 2.32)
Andean Latin America	40,909 (34,377, 48,012)	379.68 (319.06, 445.43)	1.97 (1.83, 2.12)	5,515 (3,925, 7,165)	51.30 (36.50, 66.60)	1.96 (1.82, 2.09)
Tropical Latin America	30,081 (23,322, 38,291)	61.81 (47.91, 78.66)	−1.13 (−1.27, −0.99)	3,386 (2,077, 4,928)	7.00 (4.30, 10.10)	0.32 (0.14, 0.50)
Central Latin America	69,325 (55,949, 84,914)	146.74 (118.53, 179.58)	0.27 (0.21, 0.32)	6,816 (4,407, 9,524)	14.40 (9.40, 20.10)	0.88 (0.78, 0.97)
Southern Latin America	51,225 (43,379, 59,654)	329.32 (278.60, 383.94)	1.62 (1.51, 1.73)	5,017 (3,456, 66,29)	32.40 (22.30, 42.90)	1.89 (1.72, 2.07)
Caribbean	6,286 (5,130, 7,551)	63.22 (51.64, 75.88)	1.12 (0.99, 1.25)	497 (318, 701)	5.00 (3.20, 7.10)	1.14 (1.01, 1.27)
High-income North America	439,264 (354,369, 538,694)	372.22 (299.72, 456.87)	−0.02 (−0.12, 0.08)	37,826 (23,574, 54,482)	31.80 (19.70, 45.80)	0.69 (0.51, 0.87)
Central Europe	41,195 (34,678, 48,526)	109.69 (91.75, 129.81)	0.19 (0.10, 0.28)	2,135 (1,331, 3,068)	5.80 (3.60, 8.30)	0.05 (−0.14, 0.25)
Western Europe	272,687 (224,083, 327,030)	186.84 (152.82, 224.92)	0.87 (0.68, 1.06)	24,605 (16,211, 33,914)	16.90 (11.10, 23.40)	1.59 (1.40, 1.78)
Eastern Europe	29,290 (23,409, 35,728)	45.98 (36.56, 56.34)	−2.77 (−2.88, −2.66)	1,052 (567, 1,673)	1.70 (0.90, 2.60)	−3.49 (−3.75, −3.23)
North Africaand MiddleEast	106,006 (86,692, 127,495)	116.81 (95.98, 140.03)	1.52 (1.43, 1.60)	7,252 (4,505, 10,358)	7.80 (4.90, 11.20)	1.28 (1.23, 1.33)
Western Sub-Saharan Africa	16,250 (12,744, 20,201)	41.06 (32.47, 50.74)	−0.43 (−0.51, −0.35)	1,195 (727, 1,738)	3.00 (1.80, 4.30)	−1.05 (−1.15, −0.95)
Eastern Sub-Saharan Africa	15,527 (12,011, 19,556)	46.95 (36.52, 58.87)	0.40 (0.38, 0.42)	1,375 (839, 1,997)	4.20 (2.50, 6.00)	0.13 (0.09, 0.16)
Southern Sub-Saharan Africa	15,430 (12,115, 19,400)	136.03 (106.93, 170.84)	−0.39 (−0.55, −0.22)	1,374 (838, 2,000)	12.10 (7.30, 17.60)	−0.37 (−0.55, −0.19)
Central Sub-Saharan Africa	6,792 (5,312, 8,489)	59.91 (47.19, 74.47)	0.45 (0.31, 0.59)	584 (358, 844)	5.10 (3.10, 7.40)	0.23 (0.16, 0.29)
**Location**	**Death cases (95%UI)**	**ASR per 100,000 (95%UI)**	**EAPC (95%CI)**	**DALYs (95%UI)**	**ASR per 100,000 (95%UI)**	**EAPC (95%CI)**
Global	72,574 (58,054, 86,455)	4.42 (3.53, 5.26)	0.65 (0.55, 0.75)	2,105,047 (1,737,325, 2,477,355)	128.20 (105.80, 150.80)	0.51 (0.42, 0.60)
High SDI	22,896 (21,432, 24,107)	6.10 (5.71, 6.43)	1.18 (0.94, 1.43)	662,058 (611,311, 716,200)	182.50 (168.40, 197.70)	0.96 (0.74, 1.17)
High-middle SDI	7,986 (6,747, 9,077)	2.08 (1.76, 2.37)	−0.03 (−0.12, 0.06)	247,662 (213,539, 283,921)	64.90 (56.00, 74.40)	−0.10 (−0.18, −0.02)
Middle SDI	15,215 (12,157, 19,391)	2.90 (2.32, 3.69)	0.78 (0.71, 0.86)	457,017 (375,013, 566,880)	86.00 (70.50, 106.70)	0.70 (0.63, 0.76)
Low-middle SDI	20,396 (12,914, 29,630)	7.54 (4.77, 10.95)	0.44 (0.37, 0.51)	569,963 (371,695, 809,404)	207.20 (135.00, 294.30)	0.39 (0.33, 0.44)
Low SDI	6,042 (3,321, 9,019)	6.50 (3.57, 9.72)	0.23 (0.17, 0.30)	167,217 (95,904, 245,165)	175.20 (100.40, 257.10)	0.13 (0.06, 0.19)
East Asia	3,822 (2,345, 5,483)	0.86 (0.53, 1.23)	−0.07 (−0.22, 0.08)	143,055 (103,089, 191,590)	32.20 (23.20, 43.10)	0.12 (−0.04, 0.28)
Central Asia	311 (245, 402)	1.95 (1.54, 2.51)	−2.12 (−2.58, −1.66)	10,386 (8,375, 13,120)	62.60 (50.60, 78.80)	−1.92 (−2.33, −1.49)
South Asia	29,229 (18,243, 41,993)	10.39 (6.48, 14.94)	0.20 (0.13, 0.27)	811,355 (524,667, 1,141,235)	285.10 (184.30, 401.30)	0.16 (0.10, 0.22)
Southeast Asia	909 (428, 1,996)	0.71 (0.34, 1.56)	0.02 (−0.06, 0.09)	32,849 (18,546, 62,749)	24.90 (14.10, 47.60)	0.19 (0.13, 0.24)
High-income Asia Pacific	5,434 (4,926, 5,926)	6.93 (6.27, 7.58)	0.06 (−0.16, 0.28)	158,115 (141,244, 176,883)	214.00 (190.30, 240.40)	−0.02 (−0.21, 0.16)
Oceania	76 (37, 144)	5.32 (2.60, 10.11)	0.20 (0.06, 0.33)	2,336 (1,217, 4,236)	155.60 (81.30, 282.90)	0.23 (0.10, 0.36)
Australasia	488 (418, 570)	5.02 (4.29, 5.85)	2.56 (1.91, 3.23)	13,475 (11,524, 15,593)	142.40 (121.70, 165.10)	2.36 (1.78, 2.94)
Andean Latin America	2,010 (1,472, 2,670)	18.89 (13.84, 25.08)	1.03 (0.88, 1.18)	55,104 (41,191, 71,711)	513.10 (383.70, 667.70)	1.06 (0.91, 1.21)
Tropical Latin America	1,864 (1,703, 2,021)	3.86 (3.53, 4.19)	1.66 (1.36, 1.96)	52,151 (47,853, 56,551)	107.40 (98.50, 116.50)	1.34 (1.08, 1.60)
Central Latin America	2,841 (2,539, 3,174)	6.09 (5.44, 6.80)	2.00 (1.78, 2.22)	83,022 (74,233, 92,719)	175.90 (157.30, 196.40)	1.79 (1.60, 1.98)
Southern Latin America	1,510 (1,347, 1,690)	9.53 (8.51, 10.67)	1.64 (1.37, 1.92)	42,927 (38,308, 47,869)	274.10 (244.60, 305.80)	1.56 (1.31, 1.81)
Caribbean	256 (201, 343)	2.60 (2.04, 3.49)	1.85 (1.64, 2.06)	7,422 (5,894, 9,743)	74.90 (59.50, 98.30)	1.77 (1.57, 1.97)
High-income North America	9,804 (9,262, 10,253)	7.87 (7.44, 8.23)	1.18 (0.80, 1.57)	284,399 (263,829, 305,662)	235.70 (218.40, 253.50)	0.89 (0.57, 1.21)
Central Europe	1,075 (961, 1,188)	2.64 (2.35, 2.92)	0.00 (−0.30, 0.29)	31,547 (28,180, 35,029)	81.00 (72.20, 90.10)	−0.04 (−0.30, 0.21)
Western Europe	7,846 (7,338, 8,334)	5.08 (4.76, 5.40)	2.56 (2.25, 2.88)	218,926 (202,937, 235,287)	146.30 (135.60, 157.50)	2.26 (1.97, 2.55)
Eastern Europe	680 (613, 751)	1.01 (0.91, 1.12)	−4.98 (−5.83, −4.12)	21,626 (19,386, 24,089)	33.20 (29.60, 37.00)	−4.60 (−5.33, −3.86)
North Africaand MiddleEast	1,537 (1,036, 2,301)	1.78 (1.20, 2.69)	0.78 (0.66, 0.89)	52,593 (38,372, 73,883)	59.00 (42.90, 83.20)	0.88 (0.78, 0.98)
Western Sub-Saharan Africa	1,243 (440, 2,245)	3.46 (1.22, 6.26)	−0.73 (−0.84, −0.62)	35,433 (13,816, 62,207)	94.70 (36.70, 166.80)	−0.71 (−0.82, −0.60)
Eastern Sub-Saharan Africa	762 (259, 1,594)	2.47 (0.83, 5.18)	−0.33 (−0.37, −0.28)	22,328 (8,726, 44,607)	69.80 (27.00, 140.00)	−0.28 (−0.32, −0.24)
Southern Sub-Saharan Africa	492.4 (306, 693)	4.49 (2.79, 6.30)	0.04 (−0.28, 0.36)	14,710 (9,686, 20,179)	131.30 (86.40, 179.70)	0.01 (−0.29, 0.30)
Central Sub-Saharan Africa	384 (128,947)	3.76 (1.25, 9.45)	0.07 (−0.02, 0.16)	11,287 (4,252, 26,338)	104.70 (39.10, 249.00)	0.10 (0.00, 0.19)

#### 3.1.2 Regional trends

The global burden of ILD among middle-aged and elderly populations exhibited significant regional heterogeneity in 2021. SDI stratification revealed that low-SDI regions had the lowest ASPR at 89.42 per 100,000 (95% UI: 70.54–111.70), whereas high-SDI regions showed the highest ASPR at 301.29 per 100,000 (95% UI: 244.69–367.50; Table 1). Notably, temporal trends indicated rising ASPRs across all SDI tiers, with the most pronounced increase occurring in middle-SDI regions (EAPC 0.58, 95% CI: 0.47–0.68; Table 1). Moreover, high-SDI regions not only demonstrated the highest ASPR but also accounted for the greatest absolute disease burden, with 1,068,326 prevalent cases (95% UI: 869,935–1,300,365; Table 1). Similarly, ASIR and ASDR generally increased across all five SDI regions (Table 1). High-SDI regions exhibited an ASIR of 26.00 per 100,000 (95% UI: 16.50–36.60), and notably, their incident cases nearly doubled from 36,929 (95% UI: 22,570–54,044) in 1990 to 91,851 (95% UI: 58,388–128,962) in 2021 (Table 1, Supplementary Table S2). Conversely, low-middle SDI regions showed the highest ASDR at 7.54 per 100,000 (95% UI: 4.77–10.95), while high-middle SDI regions demonstrated the lowest values for both ASIR at 8.90 per 100,000 (95% UI: 5.70–12.50) and ASDR (2.08 per 100,000; 95% UI: 1.76–2.37; Table 1). DALY analysis further revealed distinct regional patterns: low-middle SDI regions had the highest age-standardized DALY rate at 207.20 per 100,000 (95% UI: 135.00–294.30), whereas high-middle SDI regions showed the lowest rate at 64.90 per 100,000 (95% UI: 56.00–74.40; Table 1). Significantly, high-middle SDI regions were the only stratum exhibiting a declining trend in age-standardized DALY rates (EAPC−0.10%, 95% CI: −0.18% to 0.02%), suggesting potential success in ILD control within this demographic (Table 1).

**Figure 1:**
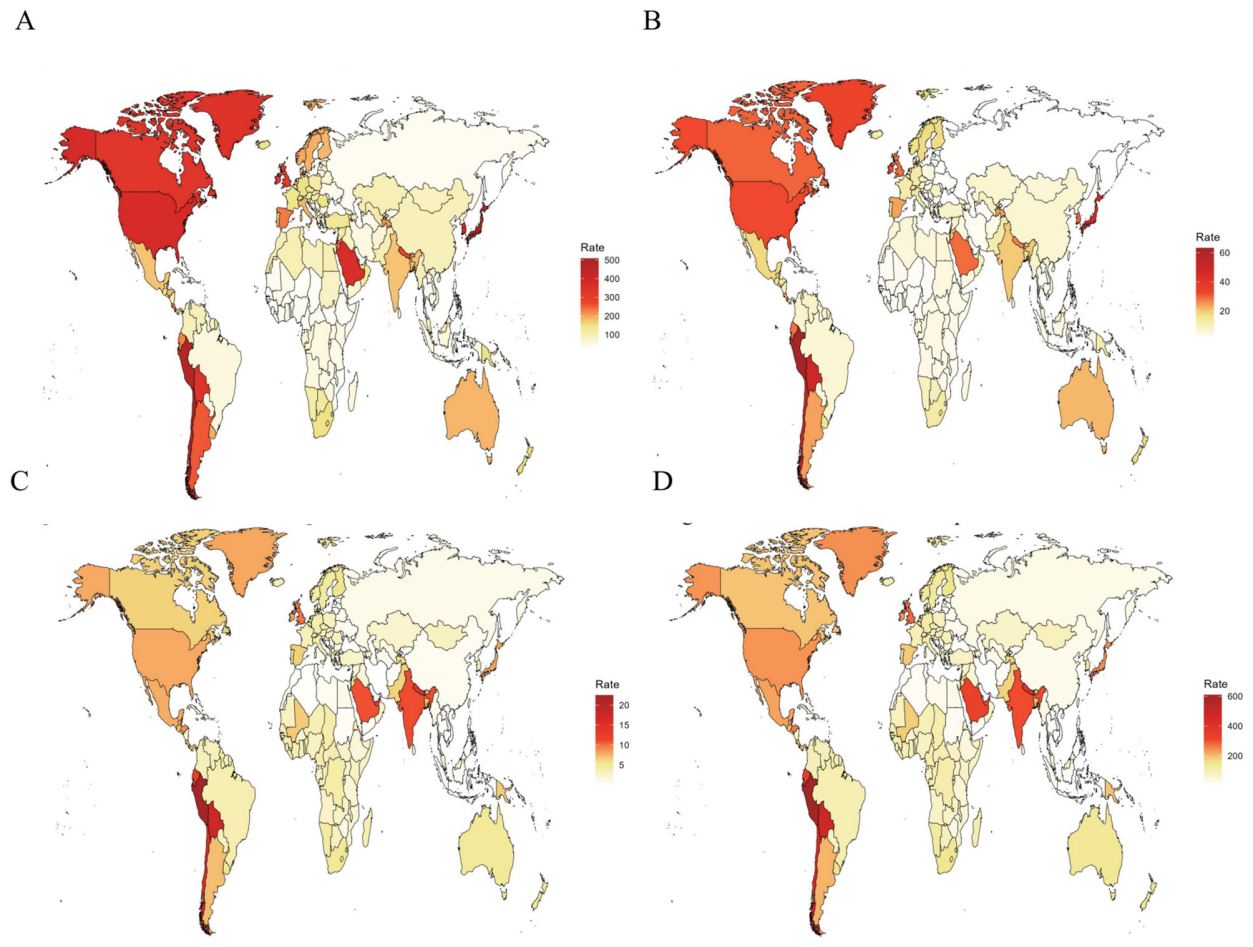
Age-standardized prevalence rate; **(B)** age-standardized incidence rate; **(C)** age-standardized death rate; **(D)** age-standardized DALYs rate. ILD, interstitial lung disease; DALYs, disability-adjusted life years.

Geographically, High-income Asia Pacific and Andean Latin America carried the highest ASPR burdens globally, with rates of 474.10 (95% UI: 384.13–579.86) and 379.68 (95% UI: 319.06–445.43) per 100,000, respectively (Table 1; Figure 1A). Regarding incidence, Andean Latin America ranked highest (ASIR 51.30 per 100,000; 95% UI: 36.50–66.60), followed by High-income Asia Pacific (41.30 per 100,000; 95% UI: 26.40–57.60; Table 1; Figure 1B). For mortality, Andean Latin America had the highest ASDR globally (18.89 per 100,000; 95% UI: 13.84–25.08), with Southern Asia ranking second (10.39 per 100,000; 95% UI: 6.48–14.94; Table 1; Figure 1C). Most notably, Andean Latin America also exhibited the world's highest age-standardized DALY rate (513.10 per 100,000; 95% UI: 383.70–667.70), substantially exceeding second-ranked Southern Asia (285.10 per 100,000; 95% UI: 184.30–401.30; Table 1; Figur 1D).

#### 3.1.3 National trends

Significant heterogeneity in ASPR was observed globally. Chile, Japan, and Peru recorded the highest ASPRs among middle-aged and elderly populations worldwide: Chile with 508.80 per 100,000 (95% UI: 429.80–596.60), Japan with 508.50 per 100,000 (95% UI: 404.90–633.00), followed closely by Peru at 468.80 per 100,000 (95%UI: 392.30–551.60). Conversely, the Philippines, Burkina Faso, and Niger showed the lowest ASPRs globally. The Philippines ranked lowest at 26.70 per 100,000 (95%UI: 19.90–34.40), with Burkina Faso following at 29.40 per 100,000 (95% UI: 22.60–36.70), and Niger at 33.30 per 100,000 (95% UI: 26.30–41.20). Notably, ASPR changes from 1990 to 2021 demonstrated substantial between-country variation (Figure 1A, Supplementary Table S3). Regarding incidence, Peru exhibited the highest ASIR at 63.30 per 100,000 (95%UI: 45.00–82.30), followed by Chile at 52.40 per 100,000 (95% UI: 36.30–70.40). Conversely, Moldova showed the lowest ASIR at 1.40 per 100,000 (95% CI: 0.70–2.10; Figure 1B, Supplementary Table S3). For mortality, Peru recorded the highest ASDR at 22.51 per 100,000 (95% UI: 14.76–32.43), while Iran demonstrated the lowest ASDR at 0.10 per 100,000 (95% UI: 0.01–0.18; Figure 1C, Supplementary Table S3). In terms of DALY burden, Peru, Mauritius, and Bolivia exhibited the highest age-standardized DALY rates: 610.7 per 100,000(95% UI: 417.4–858.0),512.4 per 100,000 (95% UI: 439.3–594.5), and 474.3 per 100,000 (95% UI: 266.9–750.0), respectively. Conversely, the Philippines and Moldova showed the lowest values at 5.9 per 100,000 (95% UI: 4.3–7.9) and 8.2 per 100,000 (95% UI: 6.0–10.6), respectively (Figure 1D, Supplementary Table S3).

### 3.2 Age and sex patterns

**Figure 2:**
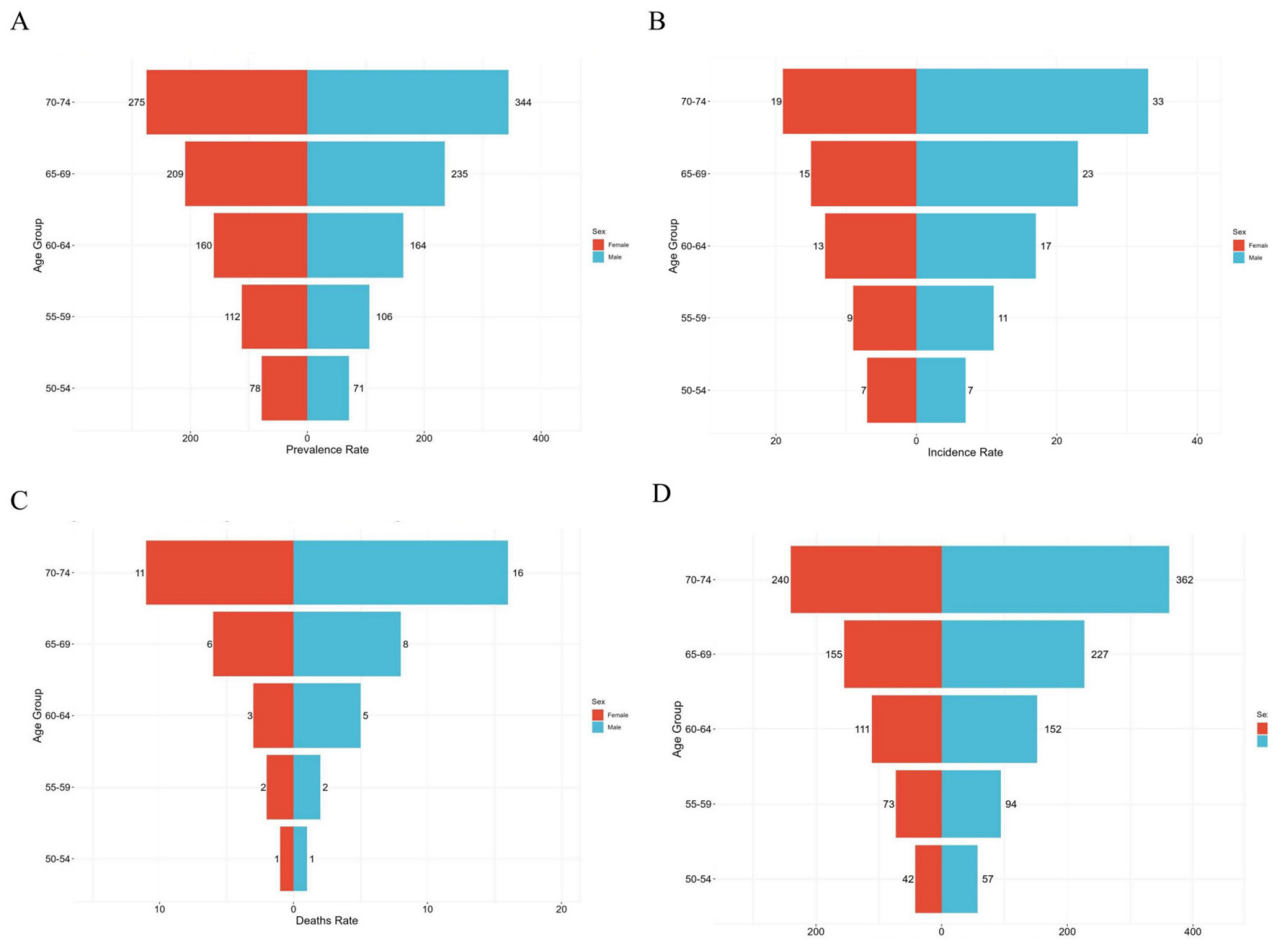
Age and Sex-structured analysis of middle-aged and older adults ILD burden in 2021. **(A)** Prevalence rate; **(B)** incidence rate; **(C)** death rate; **(D)** DALYs rate. ILD, interstitial lung disease; DALYs, disability-adjusted life years.

This report analyzes 2021 global ILD prevalence data among individuals aged 50–74 years, stratified by sex and age group. Prevalence rates with corresponding 95% confidence intervals are presented for both sexes across age strata (Figure 2). Notably, in the 50–59 age group, females exhibited slightly higher prevalence than males. However, this trend reversed with advancing age. Specifically, males surpassed females in prevalence after age 60, with the disparity widening progressively. By the 70–74 age group, male prevalence (344 per 100,000) exceeded female prevalence (275 per 100,000) by 25.1%. Moreover, prevalence increased significantly with age in both sexes (Figure 2A, Supplementary Table S4). Regarding incidence, both sexes demonstrated equal rates in the 50–54 age group. Thereafter, males showed significantly higher incidence from age 55 onward. While female incidence increased steadily with age, male incidence accelerated more rapidly, peaking in the 70–74 age group. Consistently, mortality rates were generally higher in males, with the most pronounced sex differences observed in older age groups (≥60 years) and the smallest gap in the 50–54 cohort (Figure 2B, Supplementary Table S4). Mortality demonstrated exponential age-related increases in both sexes, though the rise was more pronounced in males: men aged 70–74 experienced 16-fold higher mortality than those aged 50–54, whereas women showed an 11-fold increase (Figure 2C, Supplementary Tables S4, S8). Significantly, males exhibited higher DALY rates than females across all age groups, with this disparity widening with age and reaching its maximum in the 70–74 cohort. Furthermore, DALY rates increased substantially with advancing age in both sexes (Figure 2D, Supplementary Table S4).

### 3.3 Overall temporal trends by sex and age

**Figure 3:**
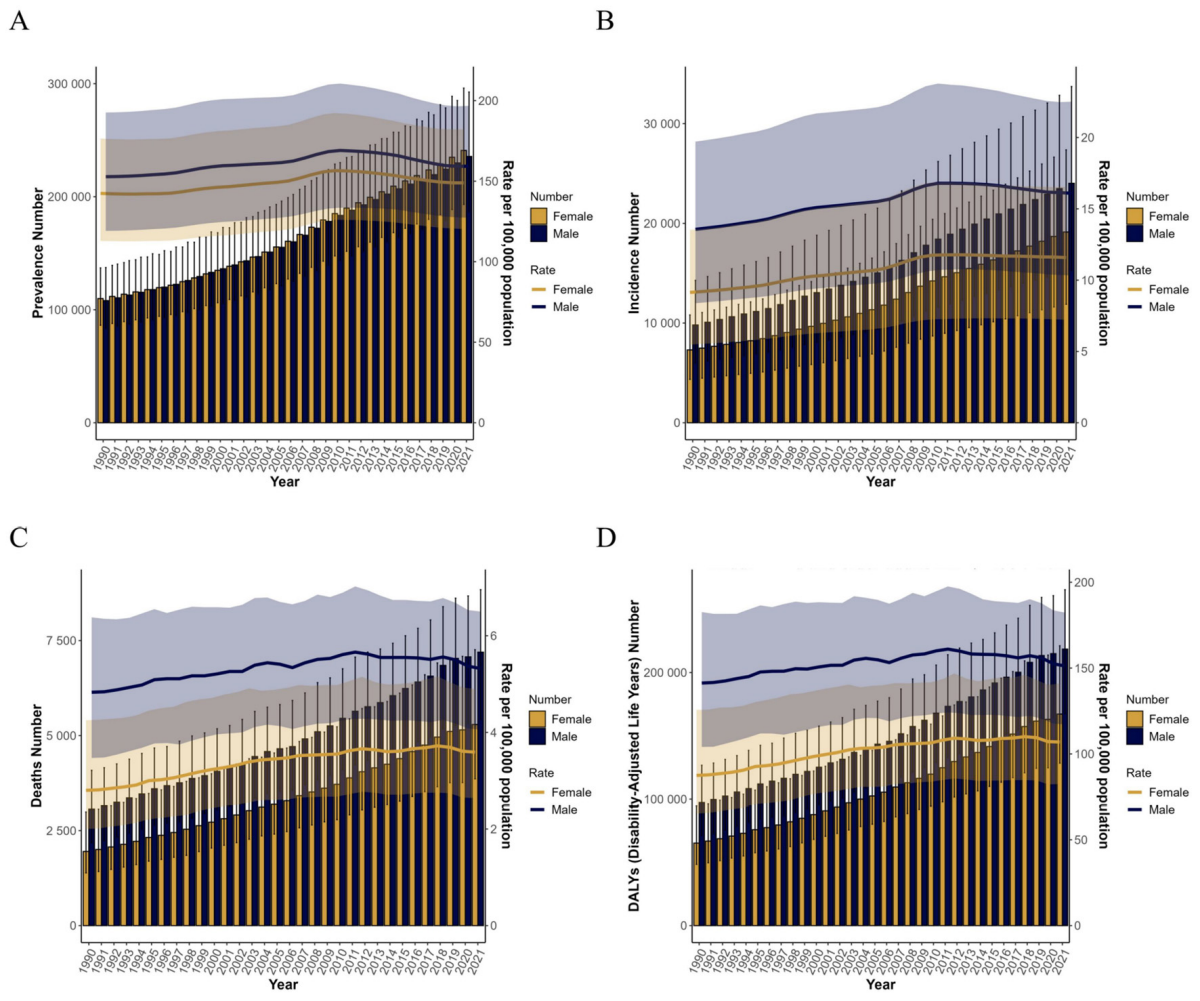
Trends in the global burden of middle-aged and older adults ILD, 1990–2021. **(A)** Prevalence rate; **(B)** death rate. **(C)** Death rate; **(D)** DALYs rate. ILD, interstitial lung disease; DALYs, disability-adjusted life years.

From 1990 to 2021, male prevalence demonstrated an initial increase from 152.78 (95% UI: 119.14–192.63) to 169.08 (95% UI: 133.53–210.58) in 2010, followed by a significant decline to 159.25 (95% UI: 127.26–196.77) by 2021. Conversely, female prevalence rose from 142.42 (95% UI: 112.90–176.49) to 156.56 (95% UI: 125.97–192.30), after which it gradually decreased to 148.93 (95% UI: 120.28–182.09; Figure 3A, Supplementary Table S5). Regarding male outcomes, incidence, mortality, and DALY rates increased from 1990 levels [13.56 (95% UI: 8.38–19.71), 4.83 (95% UI: 3.47–6.38), and 141.29 (95% UI: 104.09–182.63), respectively] to peak values in 2011 [16.79 (95% UI: 10.74–23.70), 5.66 (95% UI: 4.28–7.02), and 161.06 (95% UI: 124.23–197.56)], subsequently exhibiting declining trends. In contrast, female incidence rose from 9.14 (95% UI: 5.48–13.52) in 1990 to 11.78 (95% UI: 7.30–16.84) in 2012 before gradually decreasing. Notably, female mortality and DALY rates increased from 1990 baselines [2.80 (95% UI: 2.00–4.25) and 87.48 (95% UI: 65.30–125.67)] to 2012 peaks [3.66 (95% UI: 2.77–5.15) and 109.19 (95% UI: 85.64–146.89)], then declined, though with a transient increase in 2018 prior to subsequent reduction (Figures 3B–D, Supplementary Table S5).

### 3.4 Joinpoint regression analysis

**Figure 4:**
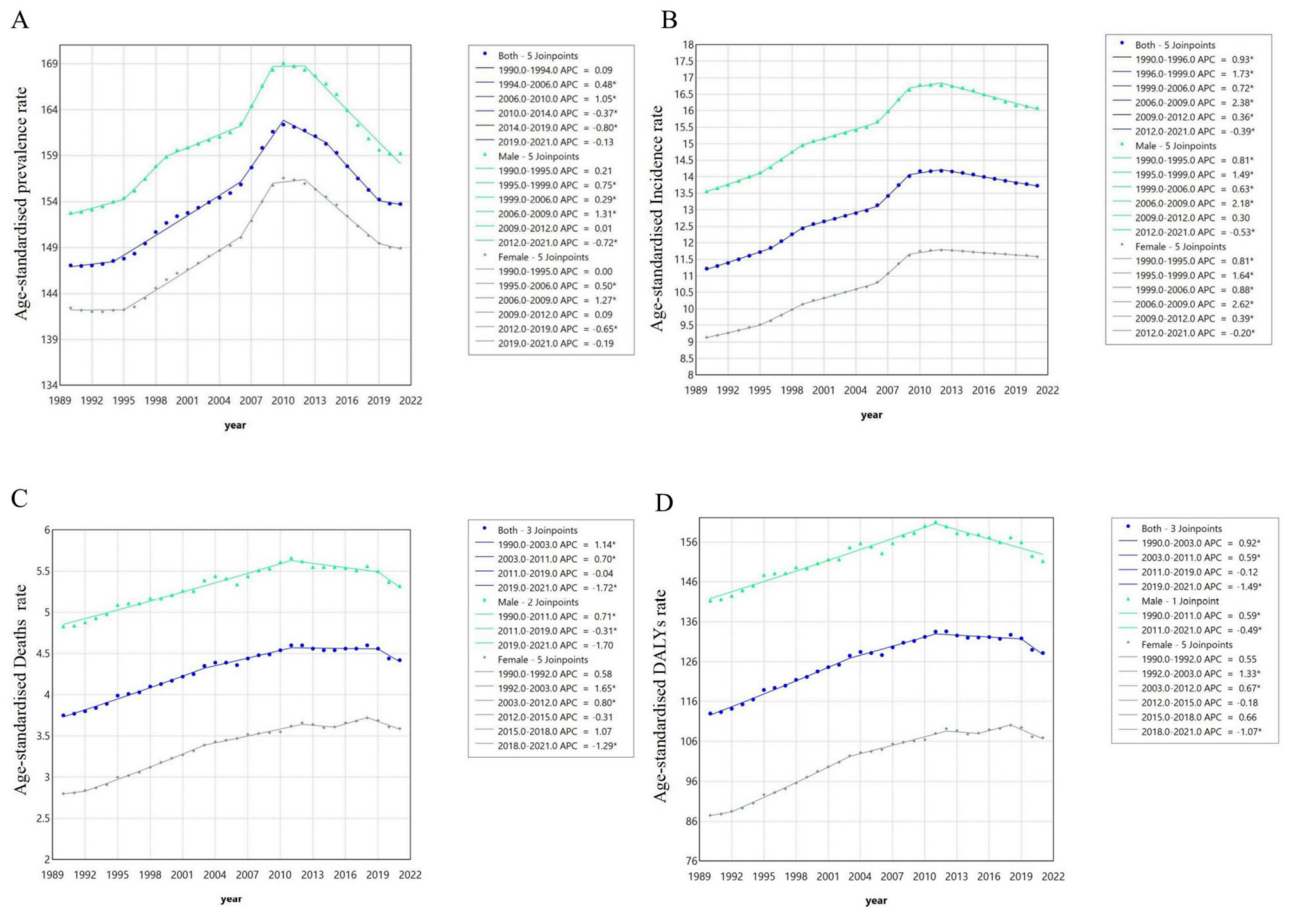
Joinpoint regression analysis of middle-aged and older adults from 1990 to 2021. **(A)** Prevalence rates; **(B)** incidence rates; **(C)** death rates; **(D)** DALYs rates.

Joinpoint regression revealed distinct temporal patterns in ILD burden metrics. For the overall population, the ASPR demonstrated an average annual percentage change (AAPC) of 0.15 (95% CI: 0.06–0.23), showing an increasing trend during 1990–2010 before transitioning to a decline in 2010–2021. Notably, a significant decreasing trend occurred between 2014 and 2019 (APC: −0.80; 95% CI:−1.01 to −0.60; Figure 4A, Supplementary Table S6). Similarly, the ASIR exhibited an overall increase-then-decrease pattern (AAPC: 0.66; 95% CI: 0.62–0.70), with modest increases during 1990–2010 followed by decreasing trends post-2010. Specifically, significant acceleration occurred during 2006–2009 (APC: 2.38; 95% CI: 2.14–2.63), while a consistent decline emerged from 2012–2021 (APC: −0.39; 95% CI: −0.41 to −0.37). The ASDR (AAPC: 0.53; 95% CI: 0.41–0.66) displayed phased transitions: a significant increasing trend during 1990–2003 (APC: 1.14; 95% CI: 1.04–1.23) was followed by attenuated growth during 2003–2011 (APC: 0.70; 95% CI: 0.49–0.91; Figure 4B, Supplementary Table S6). Remarkably, a mortality decline occurred during 2019–2021 (APC: −1.72; 95% CI: −3.18 to −0.24), potentially associated with indirect effects of the COVID-19 pandemic (Figure 4C, Supplementary Table S6). Finally, the age-standardized DALY rate (AAPC: 0.41; 95% CI: 0.31–0.51) showed significant increases during 1990–2003 (APC: 0.92; 95% CI: 0.84–1.00), with a subsequent transition to significant decline during 2019–2021 (APC: −1.49; 95% CI:−2.67 to −0.30; Figure 4D, Supplementary Table S6).

### 3.5 Frontier analysis

**Figure 5:**
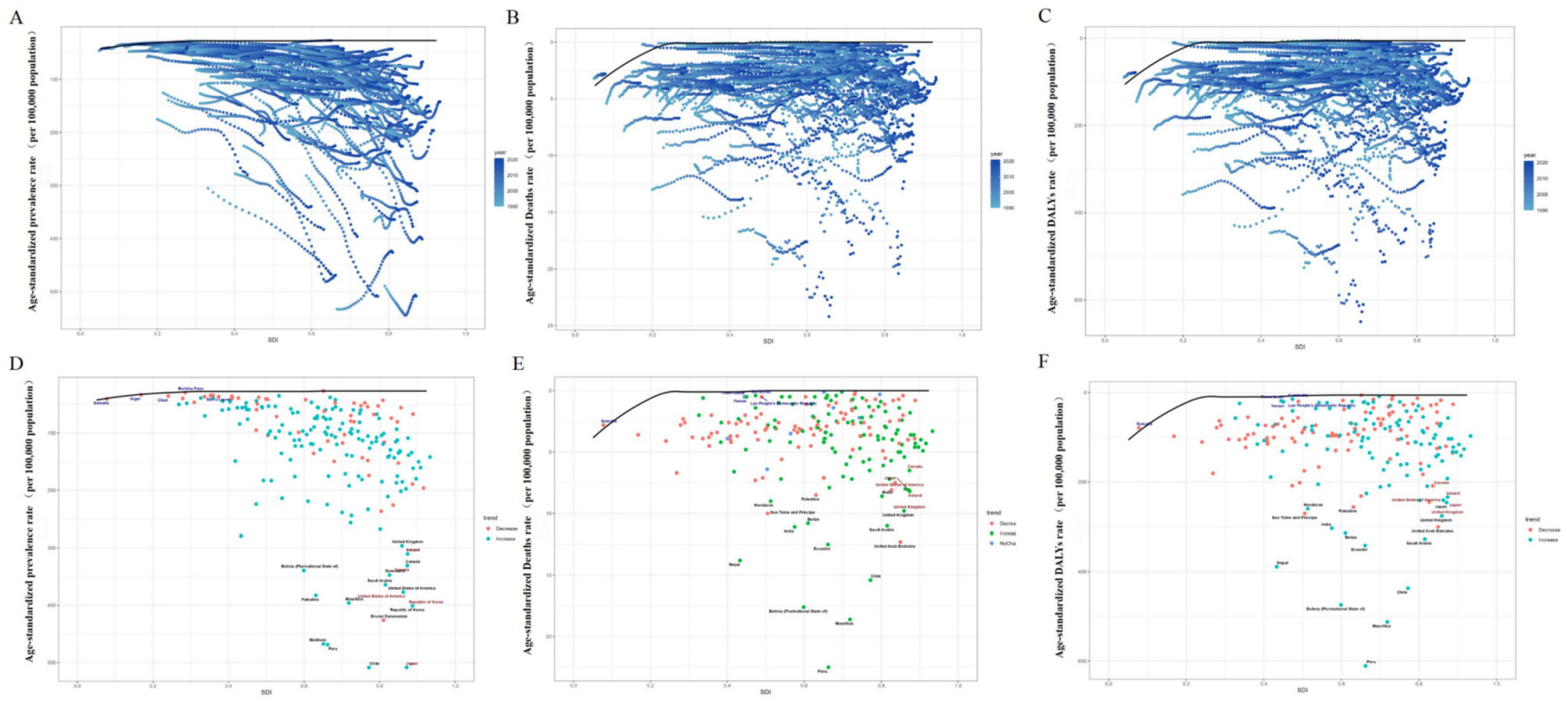
Based on SDI and the frontier analysis of ILD in middle-aged and older adults in 2021. The color change from light blue (1990) to dark blue (2021) represents the change in years. The boundaries are delineated in pure black; countries and regions are indicated by dots. The top 15 countries with the largest effective differences are marked in black; examples of frontier countries with low SDI (< 0.5) and low effective differences are marked in blue, and examples of countries and regions with high SDI (>0.85) and relatively high effective differences in their development levels are marked in red. Red dots indicate an increase in the age-standardized rate of ILD in middle-aged and elderly people from 1990 to 2019; blue dots indicate a decrease in the age-standardized rate of ILD in middle-aged and elderly people from 1990 to 2019. **(A, D)** Frontier analysis using ASPR. **(B, E)** Frontier analysis using ASDR. **(C, F)** frontier analysis using DALYS. SDI, social population index; ILD, interstitial lung disease; DALYs, disability-adjusted life years.

Frontier analysis was employed to quantify the potential for improvement in managing ILD among middle-aged and elderly populations across nations relative to their development levels. The 15 countries regions exhibiting the largest gaps between actual and attainable outcomes were Honduras, Palestine, India, Belize, Ecuador, Nepal, Chile, Bolivia, Mauritius, Saudi Arabia, United Arab Emirates, Japan, Peru, São Tomé and Príncipe, and the United Kingdom. Conversely, frontier settings with low Socio-demographic Index (SDI) included Timor-Leste, Yemen, Laos, Cambodia, and Somalia. Notably, several high-SDI countries regions—namely Canada, Ireland, the United States, Japan, and the United Kingdom—demonstrated relatively high improvement potential despite their advanced development status. Collectively, this analysis reveals substantial heterogeneity in opportunities for reducing the ILD burden across different national contexts (Figures 5A–F, Supplementary Table S7).

### 3.6 Future projections of global ILD burden in middle-aged and elderly populations

**Figure 6:**
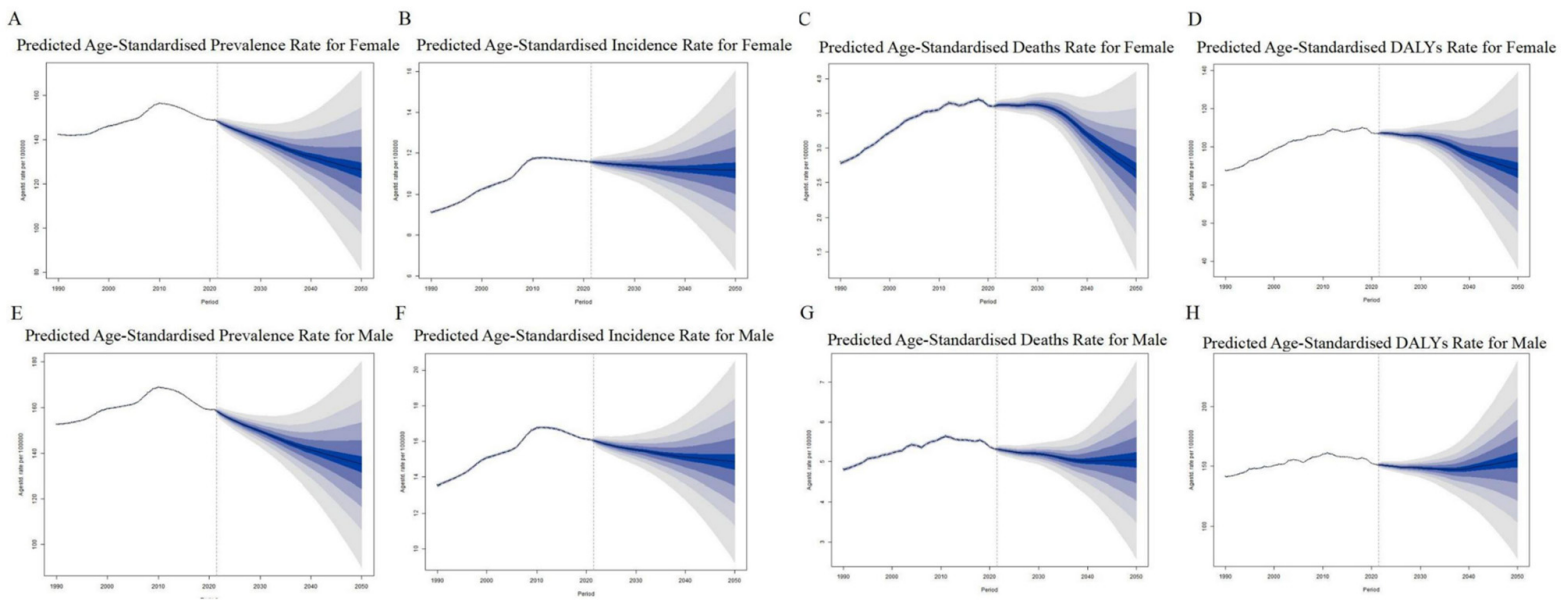
Global middle-aged and older adults ILD burden projection (2021–2050). **(A)** Prevalence rate for female; **(E)** prevalence rate for male; **(B)** incidence rate for female; **(F)** incidence rate for male; **(C)** death rate for female; **(H)** death rate for male; **(D)** DALYs rate for female; **(G)** DALYs rate for male. ILD, interstitial lung disease.

From 2021 to 2050, the ASPR is projected to decline from 149 to 142 per 100,000 females and from 159 to 152 per 100,000 males (Figures 6A, E; Supplementary Table S8). Similarly, the ASIR is expected to decrease from 12 to 11 per 100,000 in females and from 16 to 15 per 100,000 in males (Figures 6B, F; Supplementary Table S8). However, the ASDR shows divergent trends: a decrease from 4 to 2 per 100,000 females, whereas the male ASDR remains relatively stable at ~5 per 100,000 (Figures 6C, G; Supplementary Table S8). Regarding DALYs rates, females are projected to experience a reduction from 107 to 80 per 100,000, while males show a decline from 151 to 141 per 100,000 (Figures 6D, H; Supplementary Table S8). Collectively, these projections indicate that the global burden of ILD in middle-aged and elderly populations will remain substantial by 2050, with only modest declines observed across ASPR, ASIR, ASDR, and DALYs metrics.

## 4 Discussion

Against the backdrop of a rapidly aging global population, the burden of ILD among middle-aged and older adults represents a considerable public health challenge (22). Employing the GBD 2021 database, this inaugural study conducts a systematic assessment of ILD burden within the 50–74 year age demographic. We characterized the epidemiological trends of ILD from 1990 to 2021 and developed predictive models for future disease burden. Notably, our findings demonstrate that ILD continued to impose a substantial global burden in 2021, this trend is of considerable significance, primarily driven by the following factors: as demographic structure shifts, the global middle-aged and elderly population is expanding rapidly. Meanwhile, the incidence of ILD increases exponentially with age. The growing size of the high-risk population directly leads to a rise in the absolute number of cases. Additionally, the delayed effects of occupational exposures become increasingly evident in middle and old age (1). For instance, one study indicated that cumulative exposure in the construction industry is associated with a 56% increase in the risk of developing ILD (23). Therefore, against the backdrop of progressing population aging, gaining a deeper understanding of the epidemiological characteristics and driving factors of ILD in the middle-aged and elderly is of significant practical importance for developing targeted prevention and control strategies.

Among the five SDI regions, high-SDI areas exhibit a notable “double-high” pattern in ILD, characterized by the highest levels of both ASPR and ASIR globally. This phenomenon is influenced by multiple factors. Firstly, these regions generally possess well-established diagnostic systems, where widespread use of high-resolution computed tomography (HRCT) and continuous optimization of screening networks have significantly enhanced ILD detection capability, thereby contributing to the elevated reported prevalence and incidence (24). Secondly, environmental exposures accumulated through long-term industrialization in high-SDI regions further exacerbate the disease burden of ILD (25). Additionally, pronounced population aging amplifies the progression of the disease (26). It is noteworthy that although antifibrotic medications are widely accessible in these regions, substantially extending median patient survival, the severe shortage of lung transplantation resources has led to a continuous expansion of the population living long-term with the disease (27). Consequently, increased survival has not correspondingly reduced mortality. The interplay of these factors shapes the distinct and severe disease burden profile of ILD in high-SDI regions. This seemingly paradoxical coexistence of advanced healthcare and high ASPR/ASIR underscores the underlying challenges in ILD prevention and control in these areas. In contrast, low and middle-SDI regions reflect deeper socioeconomic and health-system disparities. The lack of adequate diagnosis, optimal treatment, and sufficient healthcare infrastructure likely contributes to higher ASDR and DALY rates (28). There is a particular need for these countries to promote country-specific initiatives to alleviate the burden of ILD (29).

Andean Latin America and South Asia emerge as regions of particular concern. Notably, Andean Latin America bears the most severe global burden across three dynamic indicators of ILD: incidence, mortality, and DALYs. Conversely, high-income Asia Pacific leads in prevalence—a static metric. Critically, South Asia ranks second globally for both mortality and DALY rates, thereby indicating substantial disease control challenges. Furthermore, studies demonstrate disproportionately high ILD burdens among middle-aged and elderly populations in low-middle and middle SDI regions. This disparity is likely driven by limited healthcare access and resource constraints within public health systems (30). Moreover, at the individual level, low income and lack of health insurance significantly impede access to timely diagnosis and management of ILD in middle-aged and elderly populations (31).

At the country level, Peru, Mauritius, and Bolivia recorded high age-standardized DALY rates. Bolivia and Peru, both located in South America, are notably rich in mineral resources, with Bolivia being particularly prominent in this regard. Despite their significant natural resource endowments, both countries face substantial challenges within their public health systems. Occupational exposures, especially to dust and hazardous substances in mining and related industries, contribute significantly to the burden of chronic respiratory diseases. For instance, the population attributable risk for occupational exposure in idiopathic pulmonary fibrosis (IPF) has been estimated at 26% (32). In addition to a higher burden of ILD, studies indicate that residents near the mining area of Potosí, Bolivia, face elevated risks of hypertension, hematuria, and ketonuria. Moreover, high concentrations of bioavailable lead and arsenic are present in most adobe brick houses in the region, posing ongoing health threats to the population (33). Mauritius, an island nation in Africa, has been reported to have significant indoor air pollution (34), long-term exposure to indoor pollutants has been associated with an increased likelihood of developing interstitial lung disease (4). Therefore, alongside resource development, there is an urgent need to implement effective public health interventions and environmental protection strategies to mitigate the adverse health effects of environmental pollution on local populations.

Furthermore, our study reveals distinct age and sex disparities in ILD burden among middle-aged and elderly populations. As anticipated, prevalence, incidence, mortality, and DALY rates all increase progressively with advancing age. Notably, the elevated prevalence observed in older males likely results from multifactorial influences, with differential environmental exposure histories constituting the primary determinant. Specifically, males disproportionately dominate occupations in heavy industry, manufacturing, and agriculture. Consequently, they experience substantially higher cumulative exposure to ILD-inducing agents—including dusts, fumes, and chemicals—thereby explaining their increased disease susceptibility relative to females (1). Furthermore, smoking—a key risk factor for multiple ILD—exhibits significantly higher prevalence among males than females. Consequently, this disparity substantially elevates males' risk of smoking-related pulmonary parenchymal damage (35). Moreover, emerging evidence suggests that males may experience accelerated telomere shortening, mitochondrial dysfunction, and distinct immunosenescence phenotypes during aging—intrinsic factors mechanistically linked to ILD pathogenesis (36). Finally, male patients may receive heightened clinical attention due to more pronounced symptoms or documented occupational exposure histories. Consequently, they are more likely to undergo comprehensive diagnostic evaluations for ILD, thereby potentially contributing to their observed higher confirmation rates in epidemiological statistics.

Joinpoint regression analyses identified critical inflection points in disease control trajectories. Importantly, the observed reductions in ASPR and ASIR rates likely reflect coordinated global initiatives in tobacco control, environmental health policy implementation, and population-level health education. For instance, the 2002 WHO report Reducing Risks, Promoting Healthy Life provided the first systematic quantification of environmental and behavioral risk factors, which catalyzed initiatives such as the Framework Convention on Tobacco Control and the Clean Cookstoves Initiative. These efforts directly reduced lung injury caused by smoking and exposure to biomass fuels (37)—factors known to play significant roles in the development and prognosis of ILD (38). Furthermore, bacterial, fungal, and viral infections have also been identified as important contributors to ILD (39). It has been demonstrated that viral infections, in particular, exhibit a closer association with ILD. The SARS outbreak in 2003 accelerated the modernization of global surveillance systems for respiratory infectious diseases. Through enhanced symptom screening and international reporting mechanisms by the WHO, capabilities for early intervention in ILD were indirectly improved (40). These policy measures have contributed to reducing the disease burden of ILD among middle-aged and older adults.

Frontier analyses indicate substantial unmet needs in low-SDI nations and regions. However, more notably, despite higher overall development levels, certain high-SDI regions demonstrate significant relative improvement gaps in managing ILD among middle-aged and elderly populations when benchmarked against theoretical optima. This observation implies persistent deficiencies in care delivery efficiency within these resource-advantaged settings, manifested through systemic shortcomings such as inadequate multidisciplinary collaboration, misuse of targeted therapies, and inadequate annual follow-up adherence. Collectively, frontier assessments demonstrate substantial potential for improvement in alleviating the global ILD burden among aging populations—irrespective of regional development status. Consequently, identifying regions with significant improvement potential is crucial for prioritizing resource allocation and developing targeted strategies to optimize patient outcomes.

Regarding projections, our models suggest the global ILD burden in middle-aged and elderly populations may increase further. Nevertheless, the considerable width of the 95% confidence intervals warrants cautious interpretation of these predictions.

This study holds significant clinical implications by enabling earlier detection and intervention for ILD in middle-aged and elderly populations. Given that ILD exhibits substantially elevated global prevalence, incidence, and mortality rates, it consequently presents a major public health challenge. Therefore, early identification of at-risk individuals or those in initial disease stages is imperative for implementing targeted prevention and therapeutic strategies, thereby potentially delaying disease progression and improving clinical outcomes. Furthermore, expanded epidemiological investigations are crucial to elucidate region-specific disease burden patterns and hence guide clinical prevention and control measures. Currently, epidemiological research on ILD in aging populations remains relatively limited and warrants expansion. Targeted interventions—such as environmental modifications, enhanced ILD awareness, and increased utilization of high-resolution computed tomography (HRCT) in primary care—would deepen epidemiological understanding and facilitate earlier diagnosis and treatment. Notably, establishing prioritized screening criteria is particularly critical in resource-constrained settings. Specifically, focused screening of individuals with established risk factors (e.g., smoking history, environmental exposures, or family history) could enhance cost-effectiveness and optimize resource allocation in high-burden regions with limited screening capacity. However, several methodological constraints should be considered when interpreting these findings. Since the GBD database operates at national regional levels, it precludes analysis of ethnic and racial determinants. Moreover, underdiagnosis persists substantially among elderly populations even in developed nations, compounded by heterogeneous diagnostic criteria across regions. Additionally, GBD data primarily integrate published studies and national reports rather than direct surveillance systems, which may raise concerns regarding data completeness, timeliness, and quality. Although GBD modeling improves stability through smoothing and uncertainty estimation, it cannot fully correct for systematic underdiagnosis or misclassification, thereby limiting comparability of burden estimates across regions. In the future, the focus of our work should be on obtaining more accurate and usable epidemiological data on ILD, especially in low-income regions and countries. Further research should also be conducted on the economic burden of ILD.

## 5 Conclusion

From 1990 to 2019, the global ASPR, ASIR, ASDR, and DALYs rate of ILD all showed sustained increases. High-SDI regions exhibited higher ASPR and ASIR, whereas low-middle SDI regions shouldered a heavier burden in terms of ASDR and DALYs. This disparity is likely attributable to uneven distribution of medical resources and variations in diagnostic and treatment capabilities, suggesting that low-middle SDI regions need to strengthen regulatory and policy frameworks to reduce the disease burden. Meanwhile, alongside the accelerating global aging population, gender and age have played significant roles in the burden of ILD. Although the future disease burden of ILD is projected to stabilize, it will remain at a high level. Consequently, there is an urgent need for coordinated efforts at both international and national levels to implement targeted prevention and control strategies aimed at alleviating the public health pressure imposed by ILD.

## Data Availability

The datasets presented in this study can be found in online repositories. The names of the repository/repositories and accession number(s) can be found in the article/Supplementary material.
